# HLA-B*57:01 allele prevalence in treatment-Naïve HIV-infected patients from Colombia

**DOI:** 10.1186/s12879-019-4415-3

**Published:** 2019-09-09

**Authors:** Ernesto Martínez Buitrago, José Millán Oñate, José Fernando García-Goez, Jorge Álvarez, William Lenis, Luz Marina Sañudo, Luisa Consuelo Rubiano

**Affiliations:** 1 Fundación Red de VIH del Valle del Cauca (REVIVA), Cali, Colombia; 2 Universidad del Valle, Cali, Colombia; 3 Centro Médico Imbanaco, Cali, Colombia; 4 Hospital Universitario Fundación Valle del Lili, Cali, Colombia; 5 Recuperar SA IPS, Cali, Colombia; 6 Pontificia Universidad Javeriana de Cali, Cali, Colombia; 7 Universidad Icesi, Cali, Colombia

**Keywords:** Abacavir, Antiretroviral therapy, HLA-B*57:01, HIV infection, Hypersensitivity, Pharmacogenetics. Cross-sectional study

## Abstract

**Background:**

The HLA-B*57:01 allele is associated with a hypersensitivity reaction to abacavir. Due to the lack of knowledge of HLA-B*57:01 prevalence in Colombia, routine screening is not performed and is not recommended by the national guidelines. We aimed to determine the prevalence of HLA-B*57:01 in HIV population from Colombia.

**Methods:**

This cross-sectional study included naïve HIV-infected adults from 13 cities of the country. The presence of HLA-B*57:01 was determined by using SSP-PCR in blood samples. Prevalence rates were stratified by sex, race, and region of origin.

**Results:**

HLA-B*57:01 allele prevalence in Colombian HIV-infected individuals was 2.7%. When stratifying for the race, the prevalence was 4% for whites, 2.6% for other race (mainly mestizo), and 1.9% for Afro-Colombians. The prevalence varied from 0% up to 11.4% depending on the department of origin. The highest prevalence rates were found in Caldas (11.4%), Antioquia (5%), Risaralda (4.8%), and Valle del Cauca (4.3%). When distributed by country zones, the central, with a racial predominance of Caucasians and mestizos, was the highest (6.0%, 0R = 4.1, CI 1.2–12.8, *p* = 0,016).

**Conclusions:**

The overall prevalence of HLA-B*57:01 in Colombia was lower than the reported rates for other Latin American countries such as Brazil, Costa Rica, and Argentina, but similar in comparison to Chile and Mexico. The diversity in the racial and ethnic heritage shown in our data supports the recommendation to implement routine screening for the HLA-B*57:01 allele before initiation of abacavir-containing antiretroviral therapy in the Colombian HIV management guidelines.

## Background

Abacavir, a nucleoside reverse transcriptase inhibitor (NRTI), is an antiretroviral agent frequently used as part of combination therapy for human immunodeficiency virus (HIV) infection. It is part of the first-line treatment regimens in HIV guidelines worldwide [1–3]. Although abacavir demonstrates a favorable safety profile with fewer long-term toxicities in contrast to most other NRTIs, a potentially severe hypersensitivity reaction (HSR) to abacavir has been described and strongly associated with the presence of human leukocyte antigen (HLA)-B*57:01 allele [4–6]. HSR is characterized by the presence of constitutional symptoms (fever, malaise, headache, myalgia), rash, gastrointestinal symptoms (vomiting, diarrhea), and respiratory symptoms (cough, dyspnea). These symptoms usually worsen following each consecutive dose with the risk of death if the drug is not stopped early [6, 7]. Clinical manifestations usually disappear two days after drug discontinuation. Re-exposure to abacavir could lead to severe clinical forms complications as anaphylactic reaction and death [7, 8].

HSR to abacavir occurs within six weeks of the initiation of treatment in over 90% of cases [7, 8]. The risk of developing HSR is lower among Afro-descendants, male sex, those treatment-experimented, and those with advanced HIV disease or CDC stage C (likely more experienced and therefore potentially more tolerant) [9–11]. Abacavir-related HSR had an incidence of about 5% (range 0–14%), and a mortality rate of 0.03% before the significant association with HLA-B*57:01 was established, and pretreatment genetic screening was employed [4, 8–10, 12–18]. In 2008, the PREDICT-1 study demonstrated the effectiveness of prospective HLA-B*57:01 screening in preventing abacavir-related HSR, with positive and negative predictive values of 58 and 100%, respectively [14]. Since then, current international HIV treatment guidelines recommend HLA-B*57:01 screening in HIV-infected patients at diagnosis or before initiating abacavir-containing regimens if previously unknown. Consequently, the occurrence of abacavir-related HSR has been reduced to 0–3% due to the exclusion of abacavir use in high-risk population [6, 14–18].

Prevalence of HLA-B*57:01 allele is variable among different racial and ethnic populations across the world. The Caucasians have higher prevalence rates of HLA-B*57:01 (4–8%) than African-Americans, Asians, and Hispanics (0.2–4%) [6, 14–22]. In Latin America, a few studies performed in Argentina, Brazil, Chile, Costa Rica, and Mexico have estimated the prevalence of HLA-B*57:01 allele between 2 and 5.6% among HIV-infected patients [12, 23–27]. In Colombia, however, there are no data regarding the prevalence of HLA-B*57:01 allele and current national guidelines do not consider HLA-B*57:01 screening in HIV-infected patients [3]. Colombia is a country with marked racial and ethnic differences across its geography making challenging to estimate the allele prevalence based on neighboring countries reports from the region leading this to uncertainty in the safety of the use of abacavir.

Further, the use of this pharmacogenetic test is limited by its availability and cost. The Colombian national guidelines for the management of HIV in adults and adolescents recommend the use of abacavir/lamivudine plus efavirenz as the first-line therapy and the most cost-effective regimen: however, screening for the HLA-B*57:01 allele before the initiation of treatment is not recommended for the absence of evidence-based data. Therefore, we conducted a nationwide cross-sectional study to determine the prevalence of HLA-B*57:01 allele in antiretroviral treatment-naive HIV population from Colombia.

## Methods

### Study design and population

We conducted a nationwide, multicenter, cross-sectional study which recruited HIV-infected patients receiving medical care in 14 centers for HIV comprehensive care from 13 Colombian cities (Bogotá, Medellín, Cali, Barranquilla, Montería, Cartagena, Bucaramanga, Cúcuta, Pereira, Villavicencio, Manizales, Florencia, and Pasto). From July 2017 to March 2018, treatment-naïve HIV-positive patients aged 18 years or older were consecutively included in the study, regardless of the clinical stage of HIV infection, lymphocyte (LT) CD4+ cell count or HIV viral load, time of diagnosis. We exclude patients on antiretroviral treatment or had been previously treated to avoid selection bias. All patients had been previously confirmed for HIV infection employing immunoenzymatic assays, HIV rapid tests, or Western blot tests, as defined and recommended by the clinical practice guidelines of the Colombian Ministry of Health and Social Protection and the Colombian Association of Infectious Diseases (ACIN) [3].

### Sample selection

The sample size was calculated using the proportion formula for finite populations based on an expected HLA-B*57:01 prevalence of 3% +/− 1%, an estimated population of 6000 people, a 95% confidence level, and an additional 2% to prevent patient loss. The minimum number of patients needed to estimate the prevalence of the allele was calculated as 961 patients. The cities and the corresponding sample size were defined for this study according to the regional prevalence known for Colombia for the year 2016. These cities have a population of diverse racial background and accounted for around 80% of the HIV population according to data from the Colombian non-governmental organization Cuenta de Alto Costo (CAC) [28]. Colombians are descendant from three racial groups (Caucasians, Africans, Amerindians). The ethnic composition of the Colombian population is an admixture estimated to result in mestizo (50%), Caucasian (25%), mulatto and Zambo (20%), afro-Colombian (4%), and indigenous (1%) [29].

### Data collection

Data collectors were the professionals in each institution in charge of recruiting the participants. They were trained to fill out a standardized form containing sociodemographic and clinical data, including sex, age, race, the department of origin, time since HIV diagnosis, baseline LT CD4 cell count, baseline HIV viral load, and CDC clinical stage at diagnosis. Each patient had a unique identification code used for both the case report form, and the label for a 5-mL blood sample collected after obtaining written informed consent.

Patient race was classified into four groups, Caucasians, mestizos, Afro-Colombian, and indigenous, considering the characteristics recommended in public health in Colombia, which included: characteristics of the hair, facial features, skin color, self-recognition and place of birth. The departments of origin were classified in the following zones: the Northern zone with predominance of Afro-Colombians and mestizos (departments of Atlántico, Bolívar, Sucre, Magdalena, Córdoba, Cesar, and Norte de Santander); the Central zone with predominance of Caucasians and mestizos departments of Antioquia, Caldas, Risaralda, and Quindío); The Western zone, predominance of Afro-Colombians and mestizos (departments of Chocó, Cauca, Nariño, and Valle); the Eastern (departments of Arauca, Caquetá, Casanare, Guaviare, Meta) and the Andean zones (Bogotá Distrito Especial, and departments of Cundinamarca, Huila, Tolima, Boyacá, and Santander) both with predominance of mestizos.

### Laboratory procedures

In two molecular biology laboratories (Primed Laboratory in Barranquilla and Laboratorio de Genética y Biología Molecular in Bogotá), blood samples were screened for HLA-B*57:01 carriage by using allele and group-specific polymerase chain reaction-sequence-specific primers (PCR-SSP) typing. This technique has been previously validated to be a reliable method of distinguishing between HLA-B*57:01 and other commonly occurring -B*57:02 and -B*57:03 alleles, with a sensitivity of 99,4 and 100% in two different studies, and specificity of 100% in both [30–32].

### Statistical analysis

Data describing clinical and demographic patient characteristics were summarized using medians with interquartile ranges (IQR) for continuous variables and frequencies and proportions for categorical variables. Data were entered into EpiInfo 6.04 (Centers for Disease Control and Prevention, Atlanta, USA) and then exported to Stata version 12.0 (StataCorp, College Station, TX, USA) for analysis statistical comparisons of patient characteristics by HLA-B*57:01 were made with logistic regression to evaluate the significance of differences in allelic frequencies between sex, ethnicity, and places of residence.

## Results

**Fig. 1:**
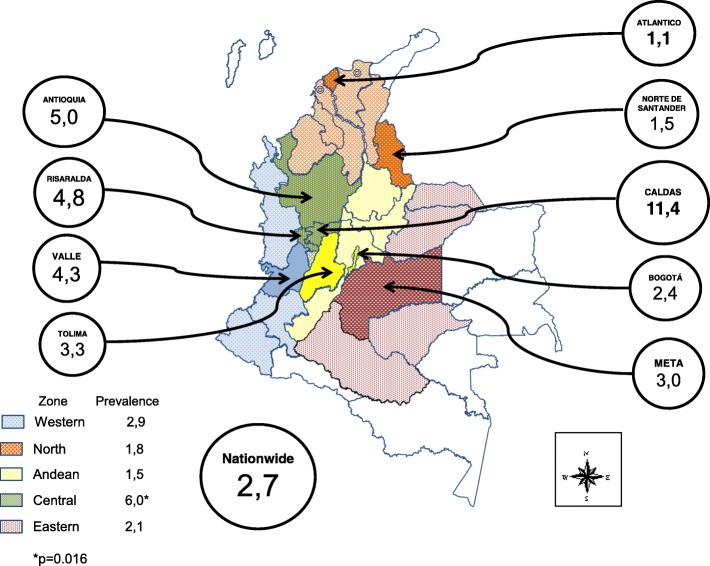
Prevalence of HLA-B*5701 Carriers In Naïve HIV-1 Infected Individuals in Colombia, by Geografic Zone (lighter color) and Department of Origin (darker color)

After exclusion of 60 patients due to missing data, a total of 902 HIV-infected patients were included in the study, of which 750 (83.1%) were male (Table 1). The median age was 29 years (interquartile range, IQR 24–39). The median time since diagnosis was 36 days (IQR 21–74). The median baseline LT CD4+ cell count and HIV viral load were 321 cells/mm3 and 44,332 copies/mL, respectively. The HLA-B*57:01 allele was found in 24 patients accounting for an overall prevalence of 2.7% (2.7% in men and 2.6% in women, *p* = 0.62) (Table 2). When stratifying for the race, the prevalence was 4% for whites, 2.6% for other race (mainly mestizo), and 1.9% for Afro-Colombians. Statistically higher positivity rates for the HLA B*57:01 allele were found among subjects with higher LT CD4 count, lower viral load, clinical stages A and B, and lower for those with an LT CD4 < 200 cells/mm^3^ and viral load > 100,000 copies/mL (Table 1). The country geographic five zones were mostly evenly represented in the sampled population, except for the Eastern zone that only had 5.3% (*n* = 48). The prevalence varied from 0% up to 11.4%, the highest prevalence rates were found in Caldas (11.4%), Antioquia (5%), Risaralda (4.8%), and Valle del Cauca (4.3%). Eighteen of the 26 regions of origin in our study population had a null prevalence. Also, when grouped by zones of the country, the prevalence ranged from 0.9% in the North Zone to 6.0% in the Central zone (Table 2, Fig. 1).

**Table 1:** Demographic, clinical and laboratory characteristics of HIV-infected patients included in the study and those positive for HLA-B*5701 allele

Variable	Total	HLA-B*5701-positive	*p*
	*N* = 902	*n* = 24	
Sex, *n* (%)			
Male	750 (83.1%)	20 (83.3%)	
Female	152 (16.9%)	4 (16.7%)	0,62
Age (years)			
Median (IQR)	29 (24–39)	36 (23–45)	
< 20	71 (7.9%)	1 (4.2%)	0,43
20–29	382 (42.4%)	9 (37.5%)	0,39
30–39	226 (26.2%)	6 (25%)	0,61
40–49	117 (12.9%)	4 (16.7%)	0,39
50–59	73 (8.1%)	3 (12.5%)	0,31
> 60	23 (2.5%)	1 (4.2%)	0,47
Race			
Afro-Colombian	54 (6.0%)	1 (4.2%)	0,58
Caucasian	50 (5.5%)	2 (8.3%)	0,39
Indigenous	5 (0.6%)	0 (0%)	0,87
Mestizo	793 (87.9%)	21 (87.5%)	0,57
Region of origin			
Andean	259 (28.7%)	4 (16,7%)	0,14
North	226 (25.1%)	2 (8,3%)	0,04
Central	200 (22.2%)	12 (50%)	0,003
Western	169 (18.7%)	5 (20,8%)	0,48
Eastern	48 (5.3%)	1 (4,2%)	0,63
Time since HIV diagnosis (days)			
Median (IQR)	36 (21–74)	67 (28–429)	
≤ 30	367 (40.7%)	8 (33.3%)	0,31
31–180	414 (45.9%)	6 (25%)	0,03
181–360	39 (4.3%)	3 (12.5%)	0,09
361–720	30 (3.3%)	3 (12.5%)	0,05
> 720	52 (5.8%)	4 (16.7%)	0,05
Baseline CD4 cell count (cells/mm^3^)			
Median (IQR)	321 (177–504)	450 (360–638)	
< 200	257 (28.4%)	2 (8.4%)	0,02
200–499	419 (46.5%)	11 (45.8%)	0,56
≥ 500	226 (25.1%)	11 (45.8%)	0,02
Baseline HIV viral load (copies/mL)			
Median (IQR)	44,332 (11206–156,278)	9064 (2749–22,248)	
< 40	14 (1.5%)	2 (8.3%)	0,06
40–1000	48 (5.3%)	3 (12.5%)	0,14
1001–10,000	145 (16.1%)	9 (37.5%)	0,01
10,001–100,000	391 (43.4%)	8 (33.3%)	0,22
> 100,000	304 (33.7%)	2 (8.3%)	0,004
CDC clinical stage at diagnosis			
A	613 (67.9%)	22 (91.7%)	0,007
B	141 (15.6%)	0 (0%)	0,02
C	148 (16.4%)	2 (8.3%)	0,22

*IQR* interquartile range

**Table 2:** Prevalence estimates for HLA-B*5701-positive stratified by sex, race, and region of origin

Variable	*n*	HLA B*5701 + (*n*)	Prevalence (%)	OR*	IC - 95%	*p*
Overall	902	24	2.7			
Race						
Mestizo	793	21	2.6	1.0		
Afro-Colombian	57	1	1.8	0.7	0.09–5.26	0.72
Caucasian	50	2	4.0	1.5	0.35–6.72	0.57
Indigenous	5	0				
Country zones and departments of origin						
Andean zone**	259	4	1.5	1.0		
Bogotá	126	3	2.4	0.7	0.07–7.04	0.77
Cundinamarca	42	0				
Tolima**	30	1	3.3	1.0		
Santander	29	0				
Boyacá	23	0				
Huila	9	0				
Central zone	200	12	6.0	4.1	1.2–12.8	0.016
Antioquia	140	7	5.0	1.5	0.18–12.9	0.69
Caldas	35	4	11.4	3.7	0.39–35.5	0.25
Risaralda	21	1	4.8	1,5	0.09–24.6	0.79
Quindío	4	0				
Western zone	169	5	2.9	1.9	0.51–7.3	0.327
Valle	118	5	4.3	1.3	0.14–11.4	0.82
Cauca	41	0				
Nariño	8	0				
Chocó	2	0				
North zone	226	2	1.8	0.6	0.10–3.13	0.518
Atlántico	87	1	1.1	0.3	0.02–5.56	0.45
Norte de Santander	65	1	1.5	0.5	0.03–7.49	0.58
Bolivar	35	0				
Magdalena	15	0				
Córdoba	13	0				
Sucre	6	0				
Cesar	5	0				
Eastern zone	48	1	2.1	1.3	0.14–12.4	0.787
Meta	33	1	3	0.9	0.05–15.16	0.95
Caquetá	10	0				
Arauca	2	0				
Casanare	2	0				
Guaviare	1	0				

*Logistic regression **reference category

The logistic regression analysis did not show any statistical association between demographic, racial, and geographical characteristics with the presence of the allele HLA-B*57:01, However, attention is drawn to the OR of the group defined as Caucasian (OR: 1.5), which loses significance due to the size of the subgroup (*p* = 0.57). On the other hand the geographical distribution according to the region where the individual was born, gains significance and confirms the tendency of the Caucasian group to show the OR: 4.1 with *p* = 0.016 for the Central area where the Caucasian ethnic group predominates, in the departments of Antioquia, Caldas, Risaralda, and Quindio (Table 2).

## Discussion

HLA-B*57:01 is a genetic marker of clinical importance that has been successfully used in several other countries, resulting in a significant decrease of abacavir-related HSR (56–100%) [14–17, 33, 34]. Ideally, since HLA-B*57:01 prevalence varies among different populations, it is essential to ascertain HLA-B*57:01 prevalence before implementing a genetic screening recommendation in a given population.

In our study, we screened Colombian HIV-infected treatment naïve patients for HLA-B*57:01 carriage. We estimated an overall HLA-B*57:01 prevalence rate of 2.7%, being lower than the reported rates for other Latin American countries such as Brazil (3.1–5.6%, *p* = 0,49 and 0,004, respectively) [12, 26], Costa Rica (5%, p = 0,07) [25], and Argentina (4.9%, *p* = 0,003) [27], but similar to those found in Chile (2.2%, *p* = 0,38) [23] and Mexico (2%, p = 0,34) [24]. The heterogeneity in HLA-B*57:01 frequency underscores the need for local studies in countries, especially those belonging to such a genetically diverse region like Latin America, as even populations with similar ancestry backgrounds might differ significantly [35]. The fact that HLA-B*57:01 prevalence for Colombia is intermediate between the frequencies reported for Caucasians and Afro-descendants is likely due to the admixture of genetic and racial backgrounds [29]. It is worth noting that consistently in our study, the prevalence rates stratified by race were 4, 2.6 and 1.9% for Whites, other races (mainly mestizo), and afro-Colombians, respectively. We found a high variability of the frequency of HLA-B*57:01 among the study regions. The lowest prevalence was observed in Atlántico (1.1%) in the Northern zone and the highest in Caldas (11.4%) in the Central zone. The North zone had the lowest rate (0.9%), with sizeable African origin inheritance, and the Central zone the highest (6.0%), with Caucasian and mestizo race predominance. While the heterogeneity of HLA-B*57:01 prevalence is mostly dependent upon the race and ethnicity heritage [6, 14–22], there are no other related factors drawn from our study data to explain the difference in the HLA-B*57:01 allele prevalence rates.

Colombia is a country with high use of abacavir-containing regimens as first-line or treatment switch (about 31%) [28], but HLA-B*57:01 is rarely performed in clinical practice. Our findings on the prevalence rates of HLA-B*57:01 confirm a substantial proportion of HLA-B*57:01 carriers in Colombia and represent the first step towards the routine genetic screening for the presence of this allele in Colombia as a measure to prevent abacavir-related HSR. Several studies elsewhere have reported HLA-B*57:01 prevalence rates from 0.3 to 7.7%, with most of them recommending screening in HIV-infected patients before using abacavir (Table 3) [12, 14, 15, 18, 21–27]. Remarkably, it must be noted that HLA-B*57:01 screening, although highly specific, never substitutes for clinical follow-up of patients starting abacavir-containing regimens [19, 36].

**Table 3:** HLA-B*5701 prevalence studies conducted in different regions across the world

Country, publication year	Number of subjects	Prevalence	HLA-B*5701 screening recommendation	Reference
Australia, 2006	260	7.7%	Yes	[15]
Taiwan, 2007	320	0.3%	No	[18]
19 countries, 2008	1956	5.6%	Yes	[14]
Chile, 2010	492	2.2%	Yes	[23]
México, 2011	300	2%	Yes	[24]
Brazil, 2011	96	3.1%	Yes	[26]
Brazil, 2014	517	5.6%	Yes	[12]
Costa Rica, 2014	200	5%	Yes	[25]
Argentina, 2015	1646	4.9%	Yes	[27]
Iran, 2016	198	3%	HLA-B*5701 may reduce risk, but there are cost issues	[22]
USA, 2017	385	3.4%	Yes	[21]

Our study had a few limitations. First, some geographic HLA-B*57:01 estimates might be over- or underestimated due to the low number of patients reported for some regions of origin. Second, HLA-B*57:01-negative patients starting abacavir-containing regimens were not followed up; therefore, the incidence of HSR was not assessed.

## Conclusions

Considering all the evidence favoring HLA-B*57:01 screening elsewhere and our findings, we strongly recommend the implementation of this pharmacogenetic test before prescribing abacavir in the HIV-infected population from Colombia. In order to determine the need for routine evaluation of HLA-B*57:01- positive individuals before initiating abacavir-containing therapy, cost-effectiveness studies could be considered in some regions of the country.

## Data Availability

The datasets generated and analyzed during the current study are available in the REVIVA (Red de VIH de Valle del Cauca) repository and are available from the corresponding author on reasonable request.

## Abbreviations

ACIN: Asociación Colombiana de Infectología
CAC: Cuenta de Alto Costo
CDC: Centers for Disease Control and Prevention
HIV: Human Immunodeficiency Virus
HLA: Human Leukocyte Antigen
HSR: Hypersensitivity Reaction
IQR: Interquartile Ranges
LT CD4 +: Lymphocyte CD4+
NRTI: Nucleoside Reverse Transcriptase Inhibitor
PCR-SSP: Polymerase Chain Reaction-Sequence-Specific Primers
